# Viruses of Fish: An Overview of Significant Pathogens

**DOI:** 10.3390/v3112025

**Published:** 2011-10-25

**Authors:** Mark Crane, Alex Hyatt

**Affiliations:** Australian Animal Health Laboratory, CSIRO Livestock Industries, Geelong Victoria 3220, Australia; E-Mail: alex.hyatt@csiro.au

**Keywords:** aquabirnavirus, betanodavirus, infectious hematopoietic necrosis virus, salmonid alphavirus, epizootic hematopoietic necrosis virus, infectious salmon anemia virus, viral hemorrhagic septicemia virus

## Abstract

The growing global demand for seafood together with the limited capacity of the wild-capture sector to meet this demand has seen the aquaculture industry continue to grow around the world. A vast array of aquatic animal species is farmed in high density in freshwater, brackish and marine systems where they are exposed to new environments and potentially new diseases. On-farm stresses may compromise their ability to combat infection, and farming practices facilitate rapid transmission of disease. Viral pathogens, whether they have been established for decades or whether they are newly emerging as disease threats, are particularly challenging since there are few, if any, efficacious treatments, and the development of effective viral vaccines for delivery in aquatic systems remains elusive. Here, we review a few of the more significant viral pathogens of finfish, including aquabirnaviruses and infectious hematopoietic necrosis virus which have been known since the first half of the 20th century, and more recent viral pathogens, for example betanodaviruses, that have emerged as aquaculture has undergone a dramatic expansion in the past few decades.

## 1. Introduction

While early studies (pre-1950s) of fish pathology indicated the presence of ‘filterable agents’, *i.e.*, viruses [1], the viral etiology of these previously recognized diseases such as infectious pancreatic necrosis, Oregon sockeye disease, although suspected, was not proven until the use of fish cell lines for the isolation of piscine viruses was established [2,3]. Over the following three decades, the availability of fish cell lines for virus isolation provided the stimulus for the development of “modern fish virology”. The ability to isolate and expand fish viruses *in vitro* not only facilitated research on viral diseases but also led to virus isolation becoming the “gold standard” for the detection of viruses in the important aquaculture species such as carp, rainbow trout and Atlantic salmon. Even in the modern molecular era virus isolation remains an important tool for research and diagnosis. Thus, over the past decade, through the use of cell culture and molecular approaches our knowledge of the biology of fish viruses has grown exponentially. Coincidentally, fish farming has expanded globally with an increase not only in absolute production (kilotonnes/year) but also in the number of fish species being cultured in both freshwater and marine systems. The increase in aquaculture operations world-wide has provided new opportunities for the transmission of aquatic viruses and the occurrence of viral diseases remains a significant limiting factor for aquaculture production and for the sustainability of biodiversity in the natural environment. Here we provide an overview of some of the significant viral pathogens affecting finfish species.

Diagnostic techniques for viral diseases of fish will not be discussed since these have become relatively standard and include pathological and histopathological examination, virus isolation on cell culture (where cell culture systems exist), molecular techniques including conventional and real-time polymerase chain reactions, *in situ* hybridization and various immunodiagnostic techniques [4].

## 2. Aquabirnavirus

*Aquabirnavirus* is the largest and most diverse of the three genera within the family *Birnaviridae*—non-enveloped viruses with a bi-segmented, double-stranded RNA genome. The type species, infectious pancreatic necrosis virus (IPNV), was the first fish virus isolated in cell culture [5] and until recently has remained one of the most intensely studied viruses of fish. The associated disease in farmed trout was recognized as early as the 1940s but was not named infectious pancreatic necrosis (IPN), based on histopathological findings, until the mid-1950s [6]. The virus isolated from a disease outbreak causing 50% mortality of rainbow trout fingerlings is the archetype IPNV strain VR-299 [1]. Since that time a vast range of IPNV and IPN-like viruses has been isolated from a very wide host range of diseased and non-diseased salmonid and non-salmonid fish species and invertebrates world-wide [7,8]. An example of a pathogenic marine aquabirnavirus (MABV) is yellowtail ascites virus (YAV), which was the first aquabirnavirus isolated from marine fish, yellowtail (*Seriola quinqueradiata*) in Japan [9]. Since then MABV have been detected in various marine hosts [10]. This diversity within the *Aquabirnavirus* genus makes classification down to the species level difficult.

IPN was first recognized as an acute contagious disease of young salmonid fry in the freshwater phase of production that could cause up to 100% mortality. More recently, disease associated with significant mortality has emerged in the post-smolt, seawater stages [11,12]. Aquatic birnaviruses that cause disease in salmonids (IPNV) are distinguished from other viruses that are serologically related to IPNV but are apparently avirulent, isolated from non-salmonid fish species and invertebrates and are named IPN-like or aquatic birnaviruses.

To date, 4 serogroups, A, B, C, D [7,13,14] have been proposed with most aquabirnaviruses comprising 9 serotypes (A1–A9) within serogroup A. These serotypes appear to correlate with geographical regions rather than host species. Viruses within the other 3 serogroups are less well studied. Genetic analysis indicates the clustering of 7 genogroups [15,16] which tend to correlate with geographical and serological characteristics [17].

**Figure 1.**
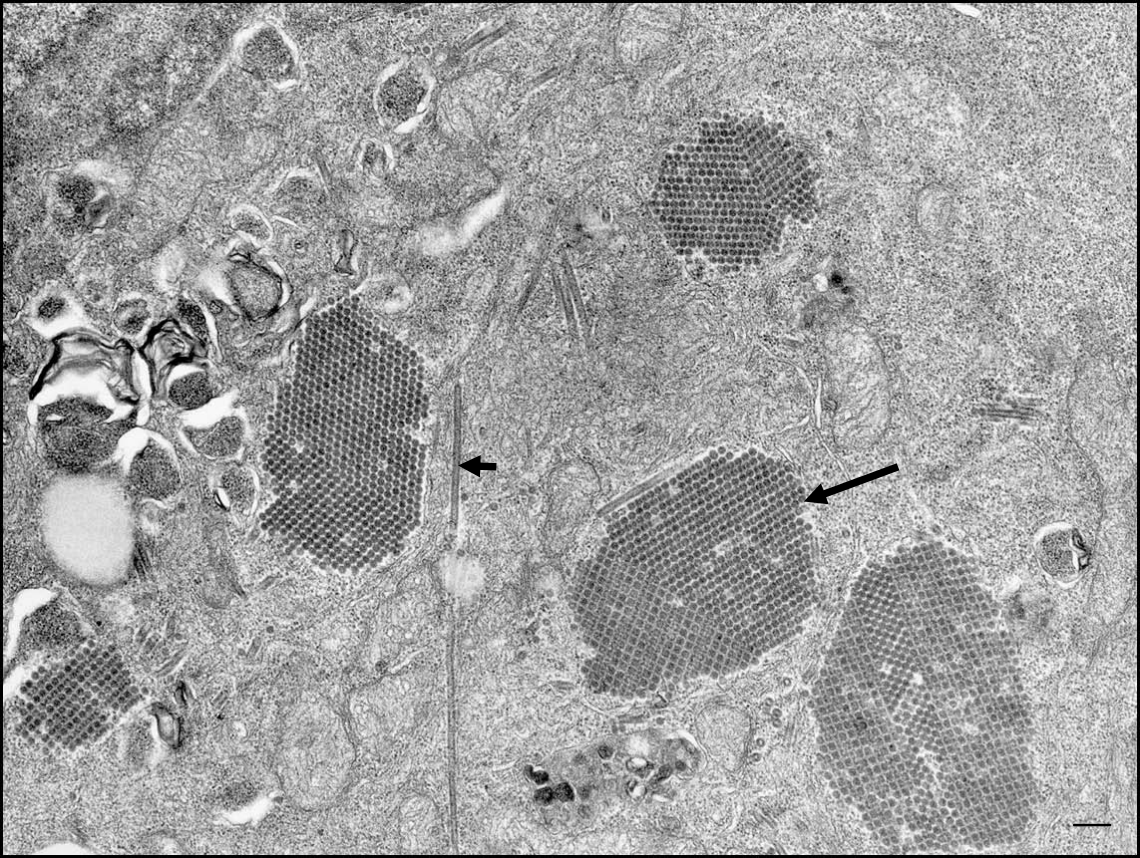
Transmission electron micrograph of a fathead minnow cell (FHM cell line) infected with infectious pancreatic necrosis virus (IPNV). Note the presence of virus-specific tubules (short arrow) and crystalline arrays of virions (long arrow). Scale bar represents 200 nm.

Aquabirnavirus particles are non-enveloped icosahedrons, 60 nm in diameter (Figure 1), containing a genome consisting of two segments (A and B) of dsRNA. Segment A encodes a polyprotein which is post-translationally cleaved to form three viral proteins VP2, VP3 and VP4, with VP2 epitopes being responsible for serotype specificity and the target for neutralizing antibodies [18]. Segment B encodes VP1, an RNA-dependent RNA polymerase [19].

Infectious pancreatic necrosis in its acute form can cause up to 100% mortality in young salmonids and remains one of the most significant diseases of major concern to the salmonid aquaculture industry. Of further significance, the majority of fish that survive a disease outbreak become sub-clinical carriers—fish that are persistently infected with no clinical signs. These sub-clinical carriers are a source of horizontal transmission, shedding virus in their feces particularly under stressful conditions such as spawning.

Mortality rates associated with disease outbreaks can be quite variable (5–100%) and it is probable that various host, viral and environmental factors influence the severity of the outbreak [20]. While some host (e.g., nutritional status) and environmental factors (e.g., water quality) can be controlled little is known about viral virulence factors [17,21–23]. The vast diversity within this group of viruses and the broad host range has hindered progress in this area.

As with all viral diseases of finfish, avoidance is an important control strategy through the use of good bio-security measures that have been developed over the decades and are now well-established [4]. However, disease outbreaks still occur and much current research is aimed at vaccine development for viral diseases of finfish including IPN. While the lack of a reliable challenge model has slowed research on immunity to IPNV infections [24–26], there are a number of vaccines available and in use [27].

## 3. Betanodavirus

*Betanodavirus* [28] is one of the two genera making up the family *Nodaviridae* and is the etiological agent of viral nervous necrosis (VNN, also known as viral encephalopathy and retinopathy—VER). The disease was first reported in barramundi (*Lates calcarifer*) farmed in Australia [29,30], Japanese parrotfish *Oplegnathus fasciatus* [31] followed a year later in turbot *Scopthalmus maximus* [32], European sea bass *Dicentrarchus labrax* [33], redspotted grouper *Epinephalus akaara* [34] and striped jack *Pseudocaranx dentex* [35]. The disease is characterized by vacuolating necrosis of neural cells of the brain, retina and spinal cord and causes up to 100% mortality in larval and juvenile fish, and can cause significant losses in older fish. The pathology has been well-described [30,36] but research on viral characterization was delayed until the virus was eventually isolated and expanded in the SSN-1 cell line [37] and since then our knowledge on the biology of this virus has expanded rapidly.

The virus infects a large range of host species—at least 40 species of marine and freshwater fish world-wide [30]—and the known host range continues to expand as new species of fish are used for aquaculture [38,39]. Of further interest is the potential of wild fish to become sub-clinical carriers as virus-contaminated water spreads from aquaculture enterprises into the marine environment particularly for those countries with large mariculture industries [40].

Viral nervous necrosis has a wide geographical distribution and includes south and east Asia, Oceania, Mediterranean Europe and Tunisia, UK, Norway and North America. To date, the virus has not been reported from South America.

**Figure 2.**
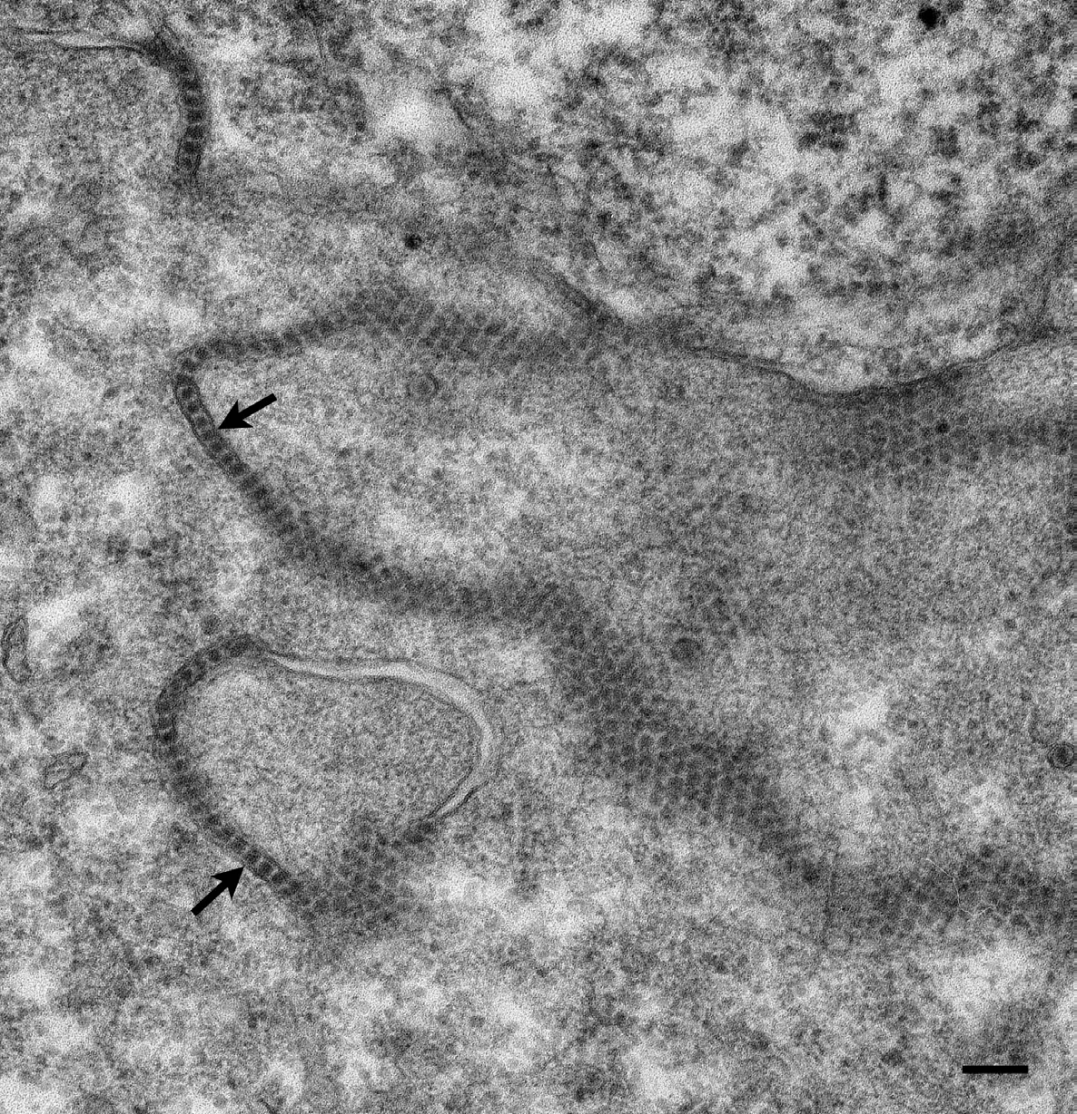
Transmission electron micrograph of an infected retinal cell with nervous necrosis virus. Virions (arrows) are located within the smooth endoplasmic reticulum. Scale bar represents 100 nm.

Virions of nervous necrosis virus (NNV) are small (25–30 nm in diameter), spherical and non-enveloped (Figure 2) with a genome consisting of two molecules (RNA1 and RNA2) of +ve sense ssRNA, the complete sequences of which have been determined [41]. RNA1 encodes a non-structural protein and RNA2 encodes the coat protein [35,42,43]. Based on the coat protein gene sequence, betanodaviruses have been classified into a number of genotypes; the number of genotypes has increased from the initially proposed four [44] to at least five genotypes [45,46], and several sub-genotypes [39], which appear to be restricted to geographical locations relating to water temperatures [47].

Current research is focused on vaccination as a potential means of control. While protection has been demonstrated in various host species using a number of different vaccination schemes [48–53], a commercially available vaccine is yet to be developed. It is likely that one vaccine will not be sufficient for the control of this disease. There are a number of considerations to be addressed including the number of different genotypes/serotypes of the virus, the relatively large number of susceptible aquaculture species, the range of environmental factors (e.g., water temperature and husbandry practices) involved with aquaculture in the different geographical locations around the world.

## 4. Infectious Salmon Anemia Virus

The orthomyxovirus, infectious salmon anemia virus (ISAV), is the causative agent of infectious salmon anemia (ISA), a disease of sea-farmed Atlantic salmon (*Salmo salar*). The disease initially reported in Norway [54] had been known for some time before its viral nature was confirmed when a new salmon cell line (SHK-1), susceptible to infection, was developed [55]. Since that time, with the ability to expand the virus *in vitro* and obtain purified virus, research accelerated and the virus is now well-characterised [56].

Since initial reports in Norway, ISA in Atlantic salmon has been reported in Canada [57–59], where it was originally designated hemorrhagic kidney disease, UK [60,61], Faroe Islands [62], USA [63] and Chile [64]. Virus has also been isolated (in the absence of ISA) from rainbow trout in Ireland [65] and Coho salmon in Chile [66].

**Figure 3.**
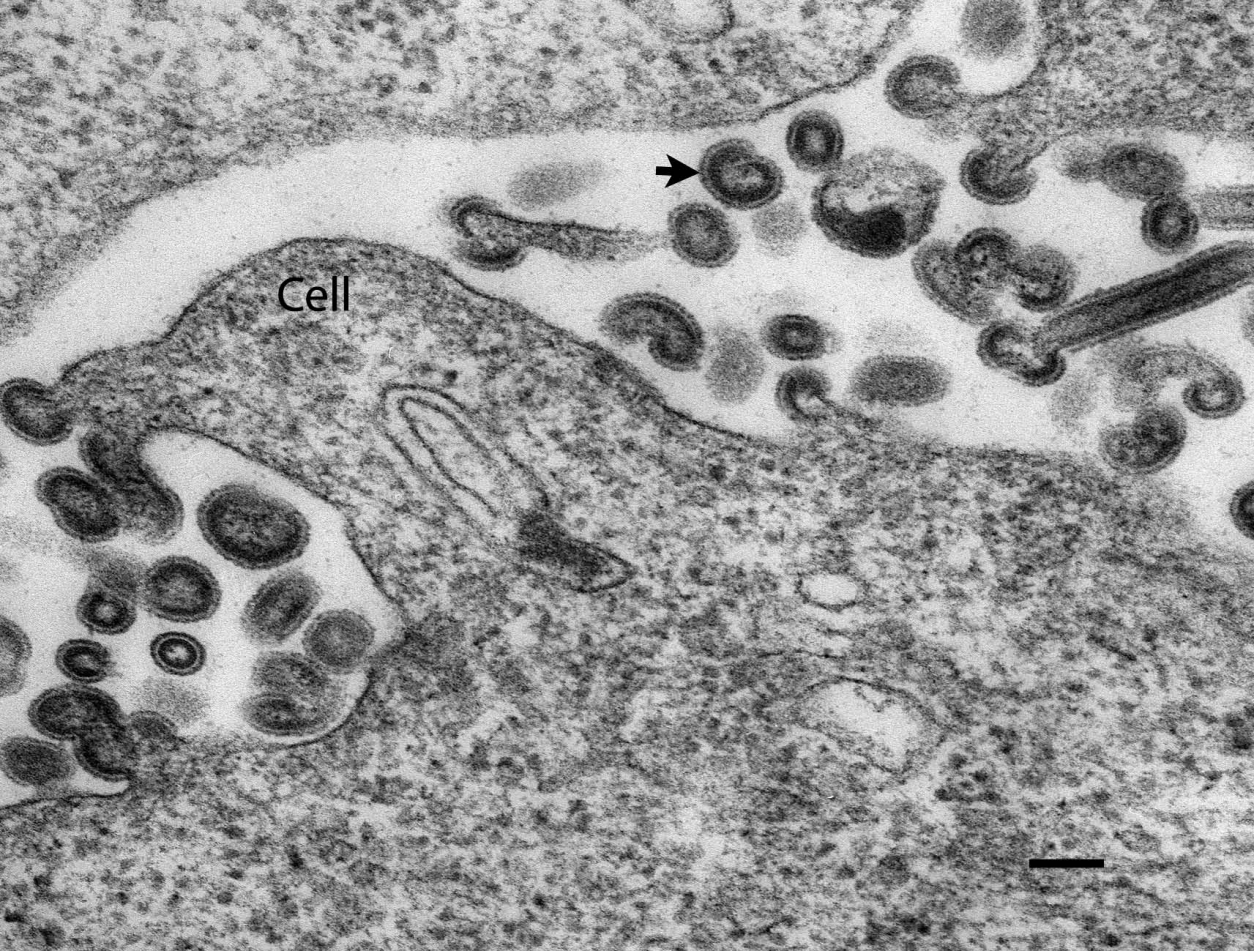
Transmission electron micrograph of a cultured cell (SHK-1 cell line) infected with infectious salmon anemia virus (ISAV). Arrow indicates an extracellular virion. Scale bar represents 100 nm.

The genetic and phenotypic characteristics of ISAV place it in the *Orthomyxoviridae* [67] and it is the type species of the genus *Isavirus* [68]. ISAV virions are pleiomorphic and enveloped with a diameter of 100–130 nm and 10–12 nm surface projections (Figure 3). The genome consists of eight negative-sense ssRNA molecules which encodes at least 10 proteins including the two main surface glycoproteins, the hemagglutinin-esterase (HE), responsible for receptor-binding and receptor-destroying activities and a fusion protein [56].

The ISAV genome has been fully sequenced [69]. Based on sequence differences of the HE gene, ISAV isolates have been divided into two major groups—the European group and the North American group [70–72]. Within these two major groups, isolates can be typed according to variations within a small, highly polymorphic region (HPR) of the hemagglutinin gene [73–76]. The HPR is characterised by the presence of gaps rather than single nucleotide mutations and, together with the fusion protein, has been implicated in viral virulence [77–80].

While fish species other than Atlantic salmon are susceptible to infection with ISAV, only Atlantic salmon develop ISA disease following infection with ISAV. Of interest, a survey of wild fish undertaken following the 1998 outbreak in farmed salmon in Scotland [60] revealed sub-clinical infections of sea trout (*S. trutta*) from which virus was isolated (5 fish positive out of 203 tested). In addition, ISAV was isolated in cell culture inoculated with one of 24 tissue pools (5 fish/pool) from cod (*Gadus morhua*) sampled from a well-boat that contained ISA-diseased salmon.

Experimental infections have demonstrated that herring (*Clupea harengus*) can be infected after immersion in ISAV-contaminated water [81], and rainbow trout (*Oncorhynchus mykiss*) by co-habitation with ISAV-infected salmon [82]. Experimental infection by intraperitoneal injection has been reported for sea/brown trout (*S. trutta*), Arctic char (*Salvelinus alpinus*) and rainbow trout [83,84]. No mortalities were associated with infection. ISAV has also been isolated from farmed Coho salmon (*O. kisutch*) in Chile [66] even though experimental studies indicate that Pacific salmon are relatively resistant to the virus [85]. Thus these fish species in which natural and/or experimental infections have been demonstrated can be considered potential carriers/reservoirs for ISAV.

As with many infectious diseases of fish, ISAV-infected fish may not necessarily exhibit clinical signs but the onset of disease may be precipitated by adverse environmental conditions or stress such as increased water temperature or poor water quality [86]. Stress factors (e.g., rising or falling temperatures) appear to play an important role in precipitating disease outbreaks in sub-clinical carrier fish; ISA outbreaks tend to occur during the spring (rising water temperatures) or with onset of winter (falling water temperatures). Cumulative mortality during an outbreak appears to be variable (from insignificant to >90%). Epidemiological studies have indicated that ISAV is transmitted from infected sources horizontally via seawater [87]. Significant risk factors include geographical proximity to infected marine sites or slaughterhouses/processing plants releasing unprocessed, contaminated water, and sharing of staff and equipment between sites. It is interesting to note that ISAV nucleic acid has been detected by RT-PCR in water samples taken up to 1.5 km away from infected sites. Thus prompt disinfection of affected and contaminated sites is likely to mitigate the risk of transmission [88].

Atlantic salmon survivors of an ISAV infection appear to be less susceptible to re-infection indicating the presence of a protective immune response [89]; in addition, convalescent antiserum has ISAV-neutralising activity [90]. For the development of vaccines the existence of different ISAV strains needs to be taken into consideration. Inactivated viral [91,92] and DNA vaccines [93] have been investigated and shown not to be 100% protective. Nevertheless, vaccines are available and have been used in North America, Faroe Islands and Norway [4].

## 5. Salmon Alphavirus

Pancreas disease (PD) has been recognized in farmed Atlantic salmon since the 1970s but the infectious nature of the disease was not proven until a toga-like virus was isolated two decades later from an outbreak in Ireland [94] and, subsequently, from outbreaks in Norway [95] and Scotland [96]. Sequence analyses [97] demonstrated that this new virus was related to the alphaviruses. Thus, Salmon alphavirus (SAV) is a relatively new species of the genus *Alphavirus* within the family *Togaviridae*. It is only recently that the relationship between salmon pancreas disease virus (SPDV) and sleeping disease virus (SDV) has been clarified. At least six subtypes of SAV (Table 1), based on analyses of partial E2 and nsP3 gene nucleotide sequences [98], have been described that are the causative agents of significant diseases of farmed Atlantic salmon and rainbow trout in Europe [99]. SAV1 or SPDV causes PD in farmed Atlantic salmon in Ireland. SAV2 or SDV causes sleeping disease in England, France, Germany, Italy, Scotland and Spain. SAV3 or Norwegian salmon alphavirus is responsible for PD in Norway, exclusively [100]. SAV4 consists of Atlantic salmon strains from Ireland, SAV5 consists of Scottish strains only and SAV6 contains one virus only, isolated from Atlantic salmon in Ireland [98].

Typical alphaviruses are transmitted by arthropods or insects, usually mosquitoes [101]. While an invertebrate vector for SAV has not been identified and direct horizontal transmission has been demonstrated [102], it is not known whether aquatic invertebrates play a significant role in the epizootiology of the SAV-associated diseases.

Alphaviruses are enveloped, spherical (*ca.* 60 nm diameter) viruses with a ssRNA genome (11–12 kb in size) the coding sequences of which are organized into two large, non-overlapping open reading frames [103]. The genomes of the reference strains of SPDV (F93-125) and SDV (S49P) have been sequenced and compared demonstrating that these strains are subtypes of the same virus [104], as indicated by an earlier comparative histopathology study [105].

Early studies suggested that pre-exposed fish developed resistance to re-infection [106]. Subsequent studies have shown that inactivated virus [107] and a recombinant, attenuated salmonid alphavirus [108] could provide good protection against PD, indicating that development of a commercial vaccine should be possible.

## 6. Infectious Hematopoietic Necrosis Virus

Infectious hematopoietic necrosis virus (IHNV) is one of three rhabdoviruses of finfish listed by the OIE (the World Organisation for Animal Health), the other two being viral hemorrhagic septicemia virus (VHSV) and spring viremia of carp virus (SVCV). It is the causative agent of infectious hematopoietic necrosis (IHN) which affects most salmonid fish species [109]. First described in the 1950s, IHNV caused severe losses in salmonid hatcheries in the Pacific Northwest of the USA and since then has spread throughout North America (USA and Canada) and overseas to Asian (PR China, Iran, Japan, Rep of Korea, Russia) and European (Austria, Croatia, Czech Republic, France, Germany, Italy, Netherlands, Poland, Slovenia, Spain) countries [4] through the movement of infected eggs and/or fish [110]. Moreover, recent studies have demonstrated that spread through Europe in recent times has occurred via trade in infected fish [111]. Following introduction to a new environment with host fish of different genetic backgrounds representing new selection pressures, studies both in Europe [111] and in Japan [112] have indicated a relatively rapid evolution of IHNV.

The virus has the typical bullet shape of members of the *Rhabdoviridae* and the genome consists of a single molecule of negative-sense ssRNA (*ca.* 11 kb) encoding six proteins: a nucleoprotein, a phosphoprotein, a matrix protein, a glycoprotein, a non-virion protein and a polymerase [113,114]. IHNV is the type species of the genus *Novirhabdovirus* which also contains VHSV.

Originally, the host range was thought to be restricted to species within the genus *Oncorhynchus*, until in 1984 when an outbreak in Atlantic salmon (*Salmo salar*) occurred for the first time [115]. Since then, in addition to most salmonid species [109], several non-salmonid species are known to be susceptible. For example, studies with white sturgeon (*Acipenser transmortanus*) demonstrated this species susceptibility to IHNV and suggested that it represented a potential virus vector [116]. Of further interest, Hirame rhabdovirus (HIRRV), a cold water virus isolated from flounder (*Paralichthys olivaceus*) and ayu (*Plecoglossus altivelis*) in Japan [117], has been shown to be closely related to IHNV and is also a member of the genus *Novirhabdovirus* [118].

The clinical signs and histopathology have been well-documented [1]. Risk factors include fish species and fish age—as with many viral diseases of finfish, younger life stages are more susceptible and losses during acute outbreaks can reach 95% [119]. There are 4 genogoups based on nucleotide sequence variation within the G gene that encodes the viral glycoprotein [112,120] and these genogroups demonstrate differential host-specificity [121] and virulence [122]. As with most other infectious diseases of farmed fish, fish density, temperature, water quality and nutritional status of the host, and virus strain influence susceptibility to IHNV [119]. Recent studies have demonstrated that the basis for viral host-specificity and virulence are, in part, due to fish host entry and kinetics of viral replication [123].

Due to the severe losses caused by IHN [109] there has been a long interest in the development of a vaccine [124–126]. While research on inactivated [127], recombinant [128] and DNA vaccines [129–131] have demonstrated that protective vaccination against IHN is readily achievable, further research is required to address practical and regulatory issues with respect to vaccine delivery and safety [132].

## 7. Epizootic Hematopoietic Necrosis Virus

Epizootic hematopoietic necrosis virus (EHNV) is a member of the genus *Ranavirus* in the family *Iridoviridae*; other genera in the family include *Iridovirus*, *Chloriridovirus, Lymphocystivirus* and *Megalocytivirus*. There are six viral species within the genus (*Ambystoma tigrinum virus; Bohle iridovirus; Epizootic hematopoietic necrosis virus; European catfish virus; Frog virus 3; Santee-Cooper ranavirus*). Species can be differentiated via RFLP profiles, virus protein profiles, DNA sequence analysis and host specificity, however none can be differentiated via classical antigen-antibody interactions (e.g., antigen capture ELISA) [4].

**Figure 4.**
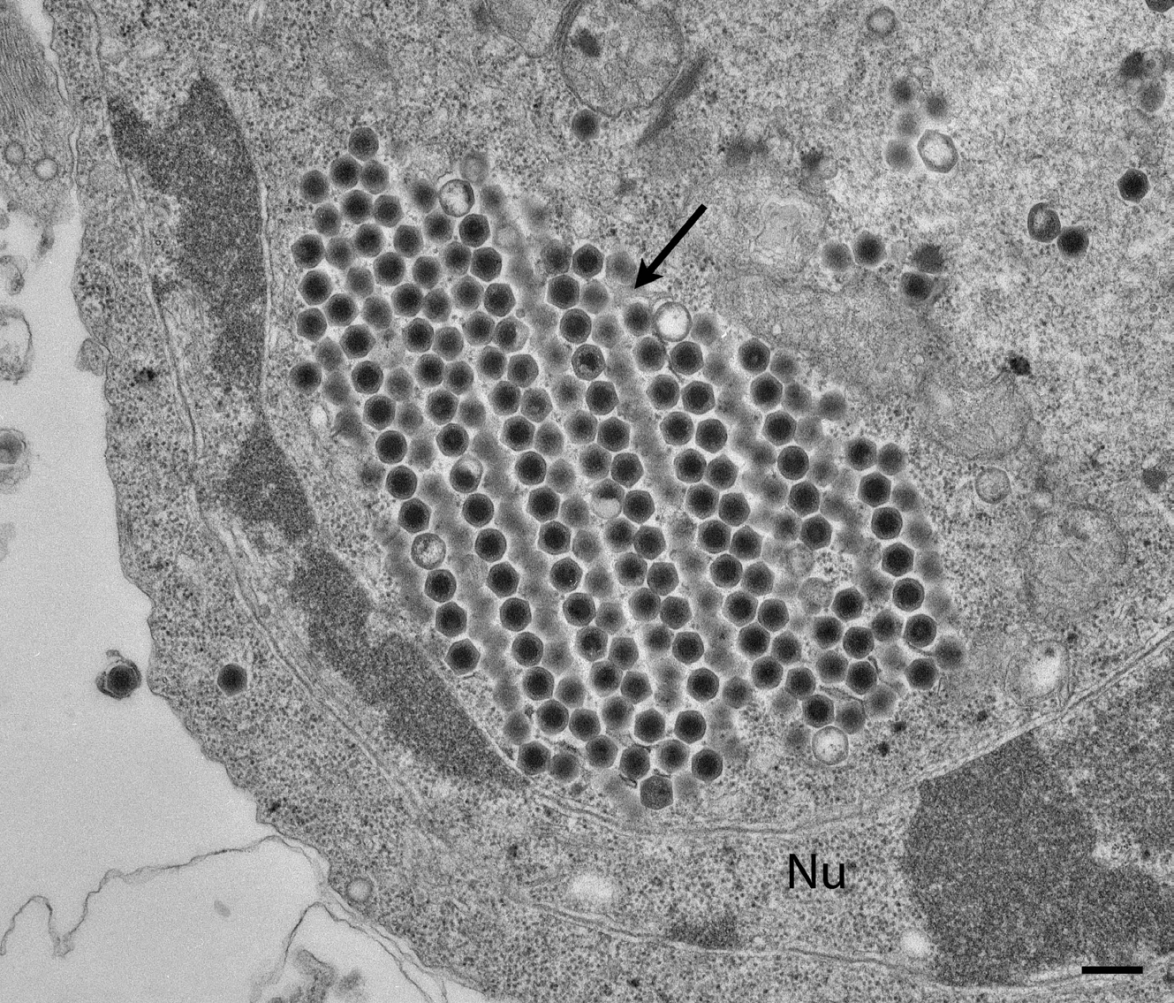
Transmission electron micrograph of a fathead minnow cell (FHM cell line) infected with epizootic hematopoietic necrosis virus (EHNV). Paracrystalline arrays are indicated by the arrow. An enveloped virus that has budded from the host cell plasma membrane can also be seen. Nu = nucleus. Scale bar represents 200 nm.

EHNV is a large icosahedral virus, approximately 175 nm (Figure 4) with a double-stranded DNA genome of 127 kb. It replicates in both the nucleus and cytoplasm with intracytoplasmic assembly. The virus obtains its outer limiting membrane via budding from the host cell plasma membrane. The inner capsid is surrounded by an internal lipid bilayer similar to that described for FV3 [133] and contains a nucleoprotein core consisting of a genome that is circularly permuted and terminally redundant.

EHNV was first isolated in 1985 from fatalities of juvenile redfin perch (*Perca fluviatilis*) in Victoria, Australia. It was the first virus isolated from freshwater finfish in Australia [134,135]. Since then it has been isolated from farmed rainbow trout (*Oncorhynchus mykiss* (Walbaum)) [136]. To date these are the only teleost species known to be naturally infected by EHNV with redfin perch being highly susceptible compared to rainbow trout which, under laboratory conditions, succumb only after intraperitoneal infection [137]. Laboratory infections by bath immersion have shown that a number of other species including Macquarie perch (*Macquaria australasica*), silver perch (*Bidyanus bidyanus*), mosquito fish (*Gambusia affinis*) and mountain galaxias (*Galaxias olidus*) are susceptible to infection and associated mortality. Other host species including Murray cod (*Maccullochella peeli*), golden perch (*Macquaria ambigua*), Australian bass (*Macquaria novemaculeata*), Macquarie perch, silver perch and Atlantic salmon (*Salmo salar*) were also shown to be susceptible following intraperitoneal injection with varying degrees of mortality [138].

The disease associated with EHNV is termed epizootic hematopoietic necrosis (EHN). The name implies the pathology of the disease includes necrosis of hematopoietic tissues. Specifically, EHNV causes multifocal necrosis of the spleen and renal hematopoietic tissue as well as the liver. Microscopically, there is a distinctive feature of infected cells—basophilic intracytoplasmic inclusion bodies—these structures are assembly sites of the virus where, by electron microscopy, paracrystalline arrays can be observed. Other lesions include hyperplasia and multifocal necrosis of gill epithelial cells, necrosis of atrial trabeculae and gastrointestinal epithelial cells, focal pancreatic necrosis, necrotic circulating hematopoietic cells and degenerate vascular endothelial cells in many organs [135,136,139–141]. Pathology such as ulcerative dermatitis and swim bladder oedema and necrosis has only been described in EHNV-infected rainbow trout [140]. EHNV infection is not associated with either enlarged/giant cells or erythrocytes.

Within Australia the geographical range is limited to south-east Australia (South Australia, New South Wales and Victoria). EHNV is endemic in these areas and is now only infrequently reported. Early work by Langdon [138] demonstrated that EHNV is extremely resistant to drying, can survive for months in water, can persist in frozen fish tissues and carcasses for at least a year [138,142]. To date, no vaccines exist for the control of EHN and so on-farm control relies on bio-security measures, health surveillance schemes and good husbandry practices that reduce physiological stressors. For infected properties, de-stocking and disinfection as per OIE protocols [4] assist in preventing re-infection.

## 7. Viral Hemorrhagic Septicemia Virus

Viral hemorrhagic septicemia virus (VHSV) is a rhabdovirus of the genus *Novirhabdovirus* (Family: *Rhabdoviridae*) and is the causative agent of viral hemorrhagic septicemia—the most serious disease of farmed rainbow trout in Europe [1]. Well-recognized since the early 1900s, VHS had been considered a disease more or less restricted to rainbow trout in Europe [1,143] until, in 1988, a VHS virus was isolated from Pacific salmon returning from the sea to two Washington State hatcheries. Subsequent genetic analyses demonstrated that this virus had not in fact been imported from Europe but was a distinct strain presumably emanating from reservoir fish species in the Pacific Ocean [144,145]. Since then, through systematic surveillance programs, the known host range for VHSV has expanded enormously to include species from several fish families including *Salmonidae*, *Esocidae*, *Clupidae*, *Gadidae*, *Pleuronectidae*, to name just a few [4]. It is likely that the true host range for this virus is so large that it will never be entirely known.

Currently, four genotypes (I-IV) and several sub-genotypes of VHSV have been identified using modern molecular tools [4]. Basically, genotype I includes the European freshwater isolates as well as some marine isolates. Genotype II consists of marine isolates from the Baltic Sea. Genotype III comprises marine isolates from the North Atlantic Ocean, and genotype IV consists of the North American (IVb) and Japanese/Korean (IVa) isolates. A separate paper in this special edition of *Viruses* is devoted to the emergence of the North American genotype IVb which has caused recent significant mortality episodes in a range of fish species inhabiting the Great Lakes of North America [146]. Therefore, the section on VHSV in this paper is only brief, understanding that there are some excellent reviews available [1,143,147,148]. Current knowledge indicates that VHSV is limited to the Northern Hemisphere, although there have been no extensive surveys of wild fish populations in the Southern Hemisphere.

It is recognized that the existence of various strains of VHSV, the large host range, and the differences in pathogenicity of these strains in the various host species presents difficulties for disease control programs. Nevertheless, it is significant that eradication of VHS post-disease outbreaks in the UK [149] and Norway [150] have been successful. Indeed, Denmark, through a persistent surveillance and control program, has been declared VHS-free, after decades with endemic VHS [151].

It was recognized early on that survivors of VHS outbreaks developed resistance to re-infection [152] and so research on vaccines against VHS has a long history [153]. Currently, while there is no available commercial vaccine, use of DNA vaccines appears promising [154].

## Figures and Tables

**Table 1.** Summary of Salmon Alphavirus (SAV) Subtypes.

**Virus Subtype**	**Host Range**	**Disease**	**Geographical Range**
SAV1	Marine Atlantic salmon	Pancreas disease	Ireland
SAV2	Freshwater rainbow trout	Sleeping disease	England, France, Germany, Italy, Scotland, Spain
SAV3	Sea-reared Atlantic salmon, rainbow trout	Pancreas disease	Norway
SAV4	Marine Atlantic salmon	Pancreas disease	Ireland, Scotland
SAV5	Marine Atlantic salmon	Pancreas disease	Scotland
SAV6	Marine Atlantic salmon	Pancreas disease	Ireland
